# Understanding arithmetic concepts: The role of domain-specific and domain-general skills

**DOI:** 10.1371/journal.pone.0201724

**Published:** 2018-09-25

**Authors:** Camilla Gilmore, Sarah Clayton, Lucy Cragg, Clare McKeaveney, Victoria Simms, Samantha Johnson

**Affiliations:** 1 Mathematics Education Centre, Loughborough University, Loughborough, United Kingdom; 2 Department of Health Sciences, University of Leicester, Leicester, United Kingdom; 3 School of Psychology, University of Nottingham, Nottingham, United Kingdom; 4 School of Psychology, Ulster University, Belfast, United Kingdom; Katholieke Universiteit Leuven, BELGIUM

## Abstract

A large body of research has identified cognitive skills associated with overall mathematics achievement, focusing primarily on identifying associates of procedural skills. Conceptual understanding, however, has received less attention, despite its importance for the development of mathematics proficiency. Consequently, we know little about the quantitative and domain-general skills associated with conceptual understanding. Here we investigated 8–10-year-old children’s conceptual understanding of arithmetic, as well as a wide range of basic quantitative skills, numerical representations and domain-general skills. We found that conceptual understanding was most strongly associated with performance on a number line task. This relationship was not explained by the use of particular strategies on the number line task, and may instead reflect children’s knowledge of the structure of the number system. Understanding the skills involved in conceptual learning is important to support efforts by educators to improve children’s conceptual understanding of mathematics.

## Introduction

A recent focus of mathematical cognition research has been to identify the cognitive skills that are related to mathematics achievement. One impetus for this work has been the belief that better understanding of the cognitive skills involved in mathematics performance can aid in identifying the key components of interventions to support mathematics learning, and help education professionals to identify children who may be at risk for difficulties in learning mathematics.

Several factors have been identified as being important for mathematics achievement. These include basic quantitative skills, such as counting, number fact knowledge and calculation skills [1–3]; accurate numerical representations, such as digit recognition, and performance on magnitude comparison and number line tasks [3–5]; and domain-general skills, including working memory, executive function and visuospatial skills [6–8].

These studies have shown that overall mathematics achievement relies on a combination of quantitative and domain-general skills. However, mathematics is a complex multi-componential skill [9] and therefore a focus solely on identifying the skills that contribute to overall achievement may fail to identify important relationships between cognitive skills and specific components of mathematics. In particular, conceptual understanding of mathematics is often considered to be a separable component from procedural and factual knowledge [10]. Procedural knowledge is defined as knowledge of an ordered sequence of steps to solve a problem, or “knowing how-to” [11–12]. For example, children need to be able to carry out addition and subtraction operations accurately and efficiently to solve arithmetical problems. In contrast, conceptual understanding is defined as understanding of the principles that underlie a domain, or “knowing why”. It is often described as a network of connections between pieces of knowledge [11–12]. For example, as well as being able to perform addition and subtraction operations, children also need to understand that addition and subtraction are inversely related. Conceptual and procedural knowledge are not hierarchically ordered and the relationship between the two may be complex [13]. Furthermore, children show individual differences in their profile of performance, such that some children may have advanced conceptual understanding despite poorer procedural skills, while others show the opposite pattern [14–16].

Studies that have explored the cognitive skills associated with mathematics achievement have largely concentrated on understanding the cognitive factors that contribute to procedural skills, and consequently we know much less about conceptual understanding [17]. Few studies have considered which basic quantitative or domain-general skills are associated with concurrent or future conceptual understanding. However, this is an important omission. Conceptual understanding is an important component of overall success with mathematics [12–13, 18–20], and mathematics educators place great emphasis on the development of conceptual understanding [21–22]. The importance of conceptual understanding was captured by a hierarchical framework proposed by Geary (see Fig 1 in [23]) in which overall mathematics achievement is viewed as the combination of the separable skills of conceptual understanding and procedural knowledge.

**Fig 1:**
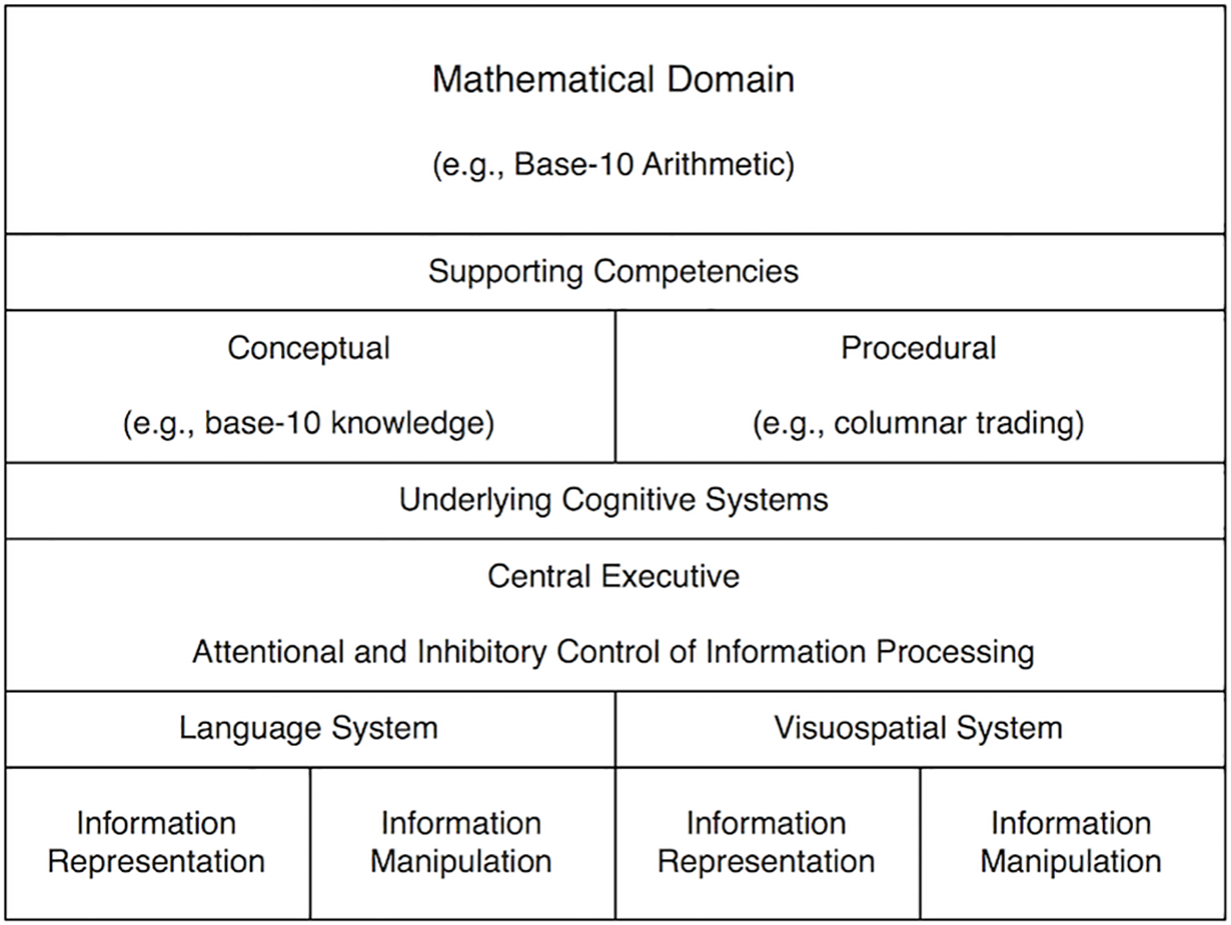
Hierarchical framework underlying mathematics achievement, adapted from Geary [23].

### The development of conceptual understanding of arithmetic

Research suggests that, before schooling, children possess rudimentary understanding of arithmetic concepts [24–27]. There is evidence that preschool children understand certain rules and procedures (e.g. a + 0 = a) even before formal education [28]. However, primary education aims to develop this early conceptual knowledge. Good conceptual understanding enables children to select appropriate mathematical procedures to solve problems and transfer understanding to both new and unfamiliar mathematical situations [28]. Within the domain of arithmetic, developing good conceptual understanding involves learning about specific principles such as commutativity, associativity, and identity, and the inverse relationships between operations (see Table 1).

**Table 1:** Key principles underlying addition and subtraction, which children need to understand for proficient arithmetic performance.

Concept	Example	Definition
Commutativity	a + b = b + a	Changing the order of addends does not change the result.
Associativity	a + (b + c) = (a + b) + c	Changing the grouping of addends does not change the result.
Identity	a + 0 = a	Adding zero to a number does not change the result.
Inversion	a + b–b = a	Addition and subtraction are inverse operations.

Developing generalized conceptual understanding of arithmetic involves a complex relationship between various specific mathematical principles [27, 29]. Currently, research has tended to investigate individual principles, with substantial focus on commutativity and inversion [30–31]. Few studies have examined more than one principle [32–34] (although see [35]), thus there are difficulties in developing a cohesive and comprehensive framework of conceptual understanding.

It has been argued that a central basis for other arithmetical concepts is the concept of additive composition, also referred to as part-whole knowledge: the principle that any natural number is the sum of other natural numbers [36]. This is fundamental to the concepts of commutativity, associativity and inversion. Emerging understanding of this concept has been linked to developments in children’s addition strategies. It has been well established that an early fundamental developmental change occurs when children transition from using “counting-all” to “counting-on” [37] strategies at around the age of 5 years-old (e.g. solving 3 + 4 by counting on from 3 rather than counting from 1). Children who use this strategy have realised that the total quantity of a set is composed of smaller quantities [38]; therefore, these children demonstrate some understanding of the additive composition of number and the number system more generally. Nunes and Bryant [39] argue a clear link between additive composition and applying an accurate understanding of the base 10 system. Whether from early exposure to the number-word system or a specific learning process, mastery of the base 10 system requires the child to have developed knowledge of additive composition [39].

Children's conceptual understanding of arithmetic continues to develop throughout schooling. By age 8 the majority of children understand the inverse principle, demonstrated by use of a shortcut strategy to solve problems of the form a + b–b [33]. However, associativity understanding develops more slowly. Only 30% of children aged 11–13 make use of an associativity-based shortcut [40]. The reasons for these individual differences in conceptual understanding have received little attention to date.

### Cognitive skills underlying conceptual understanding

Hierarchical models, such as proposed by Geary [23], highlight that conceptual understanding draws on a constellation of underlying domain-general cognitive skills, which may include attention, inhibition and verbal and visuospatial working memory. To date there has been little data available in which to test this framework and, in particular, the role of underlying cognitive skills for conceptual understanding. Of the few studies that have explored this, most have only considered working memory, with mixed results. For example, working memory, measured via a counting span task (which involves counting a sequence of sets and remembering the totals in order), was not associated with children’s understanding of counting principles [41] or conceptual understanding of fractions [42]. In contrast, working memory measured via a counting span task was correlated with conceptual understanding of fractions measured one year later [43] and conceptual understanding of multiplication was correlated with concurrent working memory measured by an operation span task, but not a simple backward digit span measure [44]. Cragg, Keeble, Richardson, Roome and Gilmore [45] found that verbal working memory (measured with a sentence span task), but not visuospatial working memory (measured with a matrix span task) was associated with conceptual understanding of arithmetic. Cowan et al. [46] found that a composite verbal working memory measure of backwards digit recall and listening recall was correlated with children’s understanding of calculation principles. Finally, Andersson [47] found that a visuospatial working memory matrix span task, but not a verbal working memory digit span task was related to understanding of calculation principles. These mixed findings suggest that both the domain of conceptual understanding measured, and the nature of the working memory assessment, may impact on findings regarding the relationship between the two. It is also notable that nearly all these studies employed verbal working memory tasks that involved numerical material or processing. There is evidence from studies exploring the relationship between working memory and mathematics achievement more generally that measures of working memory involving numerical material may inflate the relationship between working memory and mathematical skills [7]. Therefore it is important to explore the relationship between conceptual understanding and working memory using more comprehensive measures including both verbal and visuospatial working memory and tasks that do not involve numerical stimuli.

Beyond working memory, few studies have explored the relationship between broader cognitive skills, such as attention, inhibition or visuospatial skills and conceptual understanding, and most of these studies have only employed simple measures of attention or behaviour. For example, conceptual understanding of fractions has been found to be related to ratings of on-task classroom behaviour [42] as well as teacher ratings of attentive behaviour [43]. These relationships may reflect a role of executive functions such as inhibition and shifting skills, however studies involving specific cognitive measures of these skills are required to confirm this. Andersson [47] found that an experimental measure of switching was not related to conceptual understanding of arithmetic over and above general mathematics achievement. Cragg et al. [45] found that neither inhibition nor shifting were related to conceptual understanding of arithmetic in participants aged 8 to young adults. However, this study did not consider broader domain-general skills, such as visuospatial processing.

Alongside domain-general skills there is some evidence that basic quantitative skills are associated with conceptual understanding. While many studies have explored the relationship between understanding of a particular concept and use of specific counting or arithmetic procedures based on this concept [37, 48], fewer studies have explored the relationship between conceptual understanding and basic quantitative skills or numerical representations more generally. Studies have shown that young children’s understanding of arithmetic concepts is associated with basic knowledge of quantity, order, place value, number system knowledge and basic calculation skills [17, 46]. Turning to measures of numerical representations, conceptual understanding is associated with non-symbolic magnitude comparison [49] and number line performance, but not symbolic magnitude comparison [50]. Conceptual understanding is also related to more complex areas of mathematics such as problem-solving accuracy and strategy use [19].

One of the only studies to explore both quantitative and domain-general predictors of conceptual understanding investigated children’s understanding of fraction concepts. Jordan et al. [43] examined how a range of basic quantitative skills, numerical representations and domain-general skills predicted conceptual understanding measured one year later. They found that language, non-verbal IQ, teacher ratings of attention, number line task performance, calculation fluency and reading fluency were significant predictors of conceptual understanding. Number line task performance was the strongest independent predictor and the authors suggested that this may be because the number line task measures understanding of numerical magnitude and ordinality, which are important concepts for the understanding of fractions. Although working memory and non-symbolic magnitude comparison performance were correlated with later conceptual understanding, they were not significant predictors in the final multivariate model.

The study by Jordan and colleagues [43] has begun to reveal the constellation of quantitative and domain-general skills that are related to conceptual understanding, and in doing so provide evidence for Geary’s [23] framework, however it leaves many important questions unanswered. First, Jordan and colleagues explored conceptual understanding of fractions, and it is unclear whether the observed relationships also apply to conceptual understanding of arithmetic more broadly. For example, it is known that number line performance is related to procedural knowledge of fractions [51] and thus it is possible that number line performance may be specifically involved in fractions understanding rather than in conceptual understanding per se. Secondly, the study included only a single measure of working memory, which involved numerical stimuli, and no cognitive measures of broader executive functions such as inhibition and shifting or visuospatial skills. Therefore the role of domain-general skills may have been underestimated. Finally, although numerical representations were assessed using both number line and magnitude comparison tasks, the only measure of basic quantitative skills was calculation fluency. Therefore, the range of basic quantitative skills (e.g. counting, factual knowledge and strategy use and efficiency) that may be important for conceptual understanding remains to be tested.

### The current study

Here we explore the cognitive factors, both domain-general and quantitative skills, that are associated with a broad measure of conceptual understanding of arithmetic. This is not only theoretically important, to provide evidence to elaborate the framework proposed by Geary [23], but will also help to identify targets for interventions to improve children’s conceptual understanding. This was an exploratory study using data from a study of the effects of preterm birth on children’s mathematical achievement and educational outcomes [52]. Here we investigate performance on a range of mathematical and general cognitive tasks of typically developing children from the comparison group.

## Method

### Participants

Participants were 77 children (39 male) aged between 7.8 and 10.8 years (M 9.5; SD = 0.7) who formed a comparison group of typically developing children in a study of preterm birth and mathematics skills. Children were recruited from 67 mainstream schools across the East Midlands and South East of England and represented a diverse mix of socio-economic backgrounds. This sample size gives 90% power to detect a change in R^2^ of .216 (80% power to detect a change in R^2^ of .167).

### Ethical approval

Ethical approval was obtained from the Derbyshire National Health Service Research Ethics Committee and written informed parental consent was obtained for all children. Children provided verbal assent prior to taking part.

### Tasks

The children completed a battery of tasks to assess their conceptual understanding, overall mathematics achievement, quantitative skills (counting, number fact knowledge, arithmetic strategy efficiency and use, digit recognition, number line task, non-symbolic comparison and symbolic comparison), domain-general skills (working memory, inhibition, shifting and visuospatial skills) and non-verbal IQ. The conceptual understanding, counting, number fact knowledge, strategy efficiency and digit recognition tasks were adapted from Cowan et al. [46].

#### Conceptual understanding (additive composition)

Children were shown 12 pairs of arithmetic problems on a laptop screen. The answer to the first problem was given and children were asked to provide the answer to the second problem. The pairs of problems were related by one of six principles: commutativity of addition (e.g., 47 + 86 = 133; 86 + 47 = ?), subtrahend minus one (e.g., 46–28 = 18; 46–27 = ?), subtraction complement principle (e.g., 153–19 = 134; 153–134 = ?), doubles plus one (e.g., 37 + 37 = 74; 37 + 38 = ?), inverse relationship between addition and subtraction (e.g., 27 + 69 = 96; 96–69 = ?), and subtrahend plus one (e.g., 64–36 = 28; 64–37 = ?). The problems were designed to be too difficult for the children to solve via basic computation within the time limit of 10 seconds. Therefore children needed to recognize and apply the relevant principle in order to provide a correct answer within the time limit. Prior to the experimental trials children completed 4 practice trials, which involved smaller numbers and simpler principles, to ensure they understood the task. Percent accuracy scores for the experimental trials were used in the analysis. One participant did not complete this task. Cronbach’s alpha was 0.78.

#### Mathematics achievement

Children completed the Numerical Operations and Mathematics Reasoning subtests of the Wechsler Individual Achievement Test–II^UK^ [53] administered according to the standard procedure. Raw scores, combined across the two subtests, were used in the analysis. Internal reliability of these subtests for this age group is .88 for Numerical Operations and .93 for Mathematics Reasoning [53].

#### Non-verbal reasoning

Children completed the Ravens Coloured Progressive Matrices [54] administered according to the standard procedure. Standardised scores (M = 100; SD = 15) were used in the analysis. One participant did not complete this measure. Internal reliability is 0.90 [54]. We focused on nonverbal reasoning rather than verbal IQ because it is consistently found to be related to mathematics performance and some evidence suggests that there is a stronger relationship between mathematics achievement and nonverbal, compared to verbal IQ measures [55].

#### Counting

Children were asked to complete 8 ascending or descending count sequences: 25 to 32; 194 to 210; 2995 to 3004; 9996 to 10003; 46 to 38; 325 to 317; 1006 to 997; 20005 to 19998). Overall percentage accuracy was calculated. Cronbach’s alpha was 0.80.

#### Number fact knowledge

A series of 12 single-digit addition problems were read by the experimenter and children were asked to retrieve an answer as quickly as possible. Following Cowan et al. [46], the percentage of correct answers provided within 3 seconds was used as a measure of known facts. Cronbach’s alpha was 0.88

#### Arithmetic strategy use and efficiency

This task measured children’s strategy choices and efficiency when solving a total of 16 addition and subtraction problems. For each problem, children were asked to provide an answer using any strategy they wished and then to describe the strategy used. Children’s solutions and descriptions were video-recorded. Strategy efficiency was measured by recording accuracy and response times; median response time (RT) for correct problems was used in the analysis. Cronbach’s alpha was 0.91.

Children’s strategy on each trial was coded on the basis of their observable behaviors and verbal reports as retrieval, decomposition, mental counting, finger counting and other (if a child reported guessing or could not report their strategy, less than 1% of trials were coded as other). All videos were coded by two experimenters and inter-rater reliability was 94%, disagreements were resolved following discussion. The percentage of basic strategy use (mental counting, finger counting and other) was calculated and used in the analysis.

#### Number recognition

A series of 12 numbers ranging from 6 to 916 were presented on the screen and children were asked to name the number aloud as soon as they recognized it. Response times were recorded and median response time for correct trials was used in the analysis. Cronbach’s alpha was 0.79.

#### Number line

The number line task was adapted from Muldoon, Towse, Simms, Perra, and Menzies [56]. Following one practice item, children were asked to estimate the position of 22 numbers on a series of blank 0–1000 number lines. On each trial children were asked “If this is 0 and this is 1000 where would you put *N*?”. A mean score for percent absolute error (PAE) was calculated as the average distance between the actual and estimated positions of the numbers, relative to the scale of line. A sample of ten percent of the number lines were re-scored by a different experimenter, with 99.98% agreement.

#### Non-symbolic comparison

Children completed a dot comparison task (adapted from [57]) in which they were asked to select the more numerous of two simultaneously-presented arrays on a computer screen. The ratio between the dot arrays was 0.5, 0.6, 0.7 or 0.8 and the number of dots in each array varied from 5 to 28. The dot arrays were created following the method of Gebuis & Reynvoet [58] which controls for visual characteristics of convex hull and average dot size. Percentage accuracy across 80 experimental trials was calculated. Cronbach’s alpha was 0.64.

#### Symbolic comparison

Children completed a digit comparison task in which they were asked to select, as quickly as possible, the larger of two numbers presented on the screen. The trials were identical to the non-symbolic comparison task, but were presented with digits rather than dot arrays. Of the 80 trials, 4 included a 1-digit vs. 1-digit comparison, 19 involved a 1-digit vs. 2-digit comparison and 57 involved a 2-digit vs. 2-digit comparison. High accuracies are typical with this task and therefore performance was indexed via mean RT for correct trials. Cronbach’s alpha (RT) was 0.96.

#### Working memory

Working memory was assessed using three tasks, which involved different types of stimuli. 1) A backwards digit recall task in which children heard a list of digits, beginning with a span of 2, and were asked to repeat the digits in reverse order. Children received up to 6 trials per span length. They remained at each span length until they got 4 correct (and two additional marks were awarded) then moved to the next span, or discontinued if they got 3 wrong. 2) A backwards word recall task, which was identical to the backwards digit recall task but the stimuli were one-syllable animal names instead of digits. 3) The Mr X visuospatial working memory task from the standardized Automated Working Memory Assessment [59]. A composite working memory score was calculated by averaging the total scores (number of correct trials) from each of these three tasks.

#### Inhibition and switching

Inhibition and switching skills were assessed using the inhibition subtest of the NEPSY-II standardized test battery [60], which was administered following the standard procedure. Children first see a series of black and white circles and squares and are asked to name them as quickly as possible. They are then shown the items again and this time must respond with the opposite name (“circle” for squares and “square” for circles) to assess inhibition. Finally they see the shapes again and are asked to respond with the correct name for black shapes and the opposite name for white shapes to assess switching. This subtest produces standardised contrast scores (M = 10; SD = 3) for inhibition (taking into account naming performance) and switching skills (taking into account inhibition performance). Internal reliability of these subtests for this age group is 0.80 for inhibition and 0.87 for switching [60].

#### Visuo-spatial processing

Visuo-spatial processing was assessed using the Arrows subtest of the NEPSY-II standardized test battery, which was administered following the standard procedure. In this task children see a set of arrays surrounding a target and they are required to select the arrow which is pointing to the centre of the target. Standardised scores (M = 10; SD = 3) were used in the analysis. Internal reliability for this age group is 0.75 [60].

## Results

Descriptive statistics for children’s performance on each of the tasks are provided in Table 2, which demonstrate that there was good variance in performance on each task, without evidence of floor or ceiling effects. Below we first explore the relationship between conceptual understanding, quantitative and domain-general skills before going on to explore performance on the number line task in more detail. Analyses were conducted in SPSS 24.

**Table 2:** Descriptive statistics for conceptual understanding, mathematics achievement, quantitative skills, and domain-general tasks.

Task	Mean	S.D.	Min	Max
Conceptual understanding (% accuracy)	74.3	22.9	8.33	100
Mathematics achievement (standardised composite score)	103.6	20.7	53	151
Counting (% accuracy)	72.7	27.0	0	100
Number fact knowledge (% accuracy)	46.9	30.2	0	100
Arithmetic strategy use (% basic strategies)	33.8	33.4	0	100
Arithmetic strategy efficiency (median RT, seconds)	4.51	2.62	1.22	14.2
Number recognition (median RT, seconds)	0.89	0.17	0.64	1.51
Number line (percent absolute error [PAE])	8.70	6.82	2.43	35.1
Non-symbolic comparison (% accuracy)	78.7	6.61	60.0	93.8
Symbolic comparison (mean RT, seconds)	0.87	0.21	0.52	1.76
Non-verbal reasoning (standardised score)	106.3	17.1	65	135
Working memory (composite score)	13.6	3.84	6.33	25.0
NEPSY-II Inhibition (standardised contrast score)	9.71	3.40	2	16
NEPSY-II Switching (standardised contrast score)	10.9	2.89	5	17
NEPSY-II Visuo-spatial processing (standardised score)	10.4	3.15	1	18

RT = Reaction time

### The relationship between quantitative skills, domain-general skills and conceptual understanding

Conceptual understanding scores were strongly related to overall mathematics achievement (*r* = .696, *p* < .001), and this association remained significant after controlling for non-verbal IQ (*r* = .476, *p* < .001). Conceptual understanding was not associated with age (*r* = .138, *p* = .235).

Children’s conceptual understanding was correlated with all of the specific measures of quantitative skills and numerical representations (Table 3; full correlation table in S1 Table). Apart from the magnitude comparison tasks these relationships were strong (*r*’s = .55 to .68) and remained significant after controlling for non-verbal IQ. The magnitude comparison tasks had small but significant zero-order correlations, however these were no longer significant after controlling for non-verbal IQ.

**Table 3:** Correlations between conceptual understanding and quantitative skills and domain-general skills.

Task	Zero-order correlation	Controlling for non-verbal IQ
Counting (% accuracy)	.592***	.344**
Number fact knowledge (% accuracy)	.603***	.423***
Arithmetic strategy use (% basic strategies)	-.592***	-.427***
Arithmetic strategy efficiency (RT)	-.606***	-.397**
Number recognition (RT)	-.546***	-.396**
Number line (PAE)	-.675***	-.489***
Non-symbolic comparison (% accuracy)	.315**	.196
Symbolic comparison (RT)	-.263*	-.113
Working memory (composite)	.463***	.238*
NEPSY-II Inhibition (SS)	.391***	.154
NEPSY-II Switching (SS)	.327**	.162
NEPSY-II Visuo-spatial processing (SS)	.299**	.090

* p < .05

** p < .01

*** p < .001

RT = reaction time; PAE = percent absolute error; SS = standardised score.

There were moderate zero-order correlations between conceptual understanding and domain-general skills (*r*’s = .30 to .46; Table 3). Only the association with working memory remained significant after controlling for non-verbal IQ. Previous research has indicated that the nature of the working memory task may affect the relationship between working memory and conceptual understanding [44]. Therefore, we also explored the correlation with conceptual understanding for each working memory task separately. This revealed significant correlations of a similar magnitude between conceptual understanding and all three measures of working memory (Digit recall: *r* = .400, *p* < .001; Word recall: *r* = .387, *p* = .001; Mr X: *r* = .324, *p* = .004). Therefore, the composite working memory score was entered into subsequent analyses.

To investigate independent relationships between these skills and conceptual understanding, we conducted a hierarchical linear regression with conceptual understanding as Dependent Variable (DV) and all quantitative and domain-general scores as Independent Variables (IVs). Following the hierarchical model of Geary [23] we added the domain-general and domain-specific predictors in separate steps. In Model 1a (Table 4), the domain-general predictors were added in step 1 and the quantitative predictors were added in step 2. In Model 2a (Table 5) the order of the steps was reversed. To confirm that the results were specific to conceptual understanding and not simply identifying predictors of any mathematical outcome, we re-ran these models with overall mathematics achievement as DV (Model 1b Table 4 and Model 2b Table 5; see also S2 Table with separate mathematics achievement subtests as DV).

**Table 4:** Hierarchical linear regression predicting conceptual understanding (Model 1a) and overall mathematics achievement (Model 1b) by quantitative and domain-general skills.

		Model 1a DV: conceptual understanding			Model 1b DV: mathematics achievement		
Step	Predictor	β	*t*	*p*	β	*t*	*p*
1	Age (months)	.137	1.185	.240	.256	2.275	.026
2	Age (months)	.147	1.362	.178	.205	2.315	.024
	Working memory (composite)	.253	2.030	.046	.508	4.944	< .001
	NEPSY-II Inhibition (SS)	.281	2.525	.014	.282	3.094	.003
	NEPSY-II Switching (SS)	.206	1.829	.072	.151	1.632	.107
	NEPSY-II Visuo-spatial processing (SS)	.020	.165	.870	-.074	-.743	.460
3	Age (months)	-.094	-.925	.359	.049	.776	.441
	Working memory (composite)	.038	.329	.743	.237	3.287	.002
	NEPSY-II Inhibition (SS)	.100	1.016	.314	.102	1.666	.101
	NEPSY-II Switching (SS)	.130	1.394	.168	.095	1.630	.108
	NEPSY-II Visuo-spatial processing (SS)	-.065	-.631	.531	-.105	-1.641	.106
	Counting (%)	.155	1.229	.224	.248	3.139	.003
	Number fact knowledge (%)	.299	1.995	.051	.326	3.459	.001
	Arithmetic strategy use (% basic)	.063	.398	.692	.009	.088	.930
	Arithmetic strategy efficiency (RT)	.116	.718	.476	-.139	-1.388	.170
	Number recognition (RT)	-.145	-1.112	.271	.003	.042	.967
	Number line (PAE)	-.462	-3.235	.002	-.104	-1.120	.267
	Non-symbolic comparison (%)	.037	.367	.715	-.055	-.875	.385
	Symbolic comparison (RT)	.102	.959	.342	-.075	-1.133	.261

Model 1a: R^2^ change for Step 1 = .019, *p* = .240; R^2^ change for Step 2 = .302, *p* < .001; R^2^ change for Step 3 = .285, *p* < .001; Total R^2^ = .606; Model 1b: R^2^ change for Step 1 = .065, *p* = .026; R^2^ change for Step 2 = .473, *p* < .001; R^2^ change for Step 3 = .306, *p* = .002; Total R^2^ = .844; RT = reaction time; PAE = Percent Absolute Error.

**Table 5:** Hierarchical linear regression predicting conceptual understanding (Model 2a) and overall mathematics achievement (Model 2b) by quantitative and domain-general skills.

		Model 2a DV: conceptual understanding			Model 2b DV: mathematics achievement		
Step	Predictor	β	*t*	*p*	β	*t*	*p*
1	Age (months)	.137	1.185	.240	.256	2.275	.026
2	Age (months)	-.134	-1.440	.155	.047	.723	.473
	Counting (%)	.196	1.609	.112	.310	3.644	.001
	Number fact knowledge (%)	.284	1.914	.060	.304	2.916	.005
	Arithmetic strategy use (% basic)	.002	.015	.988	-.126	-1.199	.235
	Arithmetic strategy efficiency (RT)	.122	.782	.437	-.144	-1.324	.190
	Number recognition (RT)	-.159	-1.246	.217	.006	.067	.946
	Number line (PAE)	-.469	-3.459	.001	-.093	-.949	.346
	Non-symbolic comparison (%)	.041	.433	.666	-.016	-.244	.808
	Symbolic comparison (RT)	.096	.919	.362	-.115	-1.609	.112
3	Age (months)	-.094	-.925	.359	.049	.776	.441
	Counting (%)	.155	1.229	.224	.248	3.139	.003
	Number fact knowledge (%)	.299	1.995	.051	.326	3.459	.001
	Arithmetic strategy use (% basic)	.063	.398	.692	.009	.088	.930
	Arithmetic strategy efficiency (RT)	.116	.718	.476	-.139	-1.388	.170
	Number recognition (RT)	-.145	-1.112	.271	.003	.042	.967
	Number line (PAE)	-.462	-3.235	.002	-.104	-1.120	.267
	Non-symbolic comparison (%)	.037	.367	.715	-.055	-.875	.385
	Symbolic comparison (RT)	.102	.959	.342	-.075	-1.133	.261
	Working memory (composite)	.038	.329	.743	.237	3.287	.002
	NEPSY-II Inhibition (SS)	.100	1.016	.314	.102	1.666	.101
	NEPSY-II Switching (SS)	.130	1.394	.168	.095	1.630	.108
	NEPSY-II Visuo-spatial processing (SS)	-.065	-.631	.531	-.105	-1.641	.106

Model 2a: R^2^ change for Step 1 = .019, *p* = .240; R^2^ change for Step 2 = .566, *p* < .001; R^2^ change for Step 3 = .021, *p* = .528; Total R^2^ = .606; Model 2b: R^2^ change for Step 1 = .065, *p* = .026; R^2^ change for Step 2 = .732, *p* < .001; R^2^ change for Step 3 = .048, *p* = .002; Total R^2^ = .844; RT = reaction time; PAE = Percent Absolute Error.

**Fig 2:**
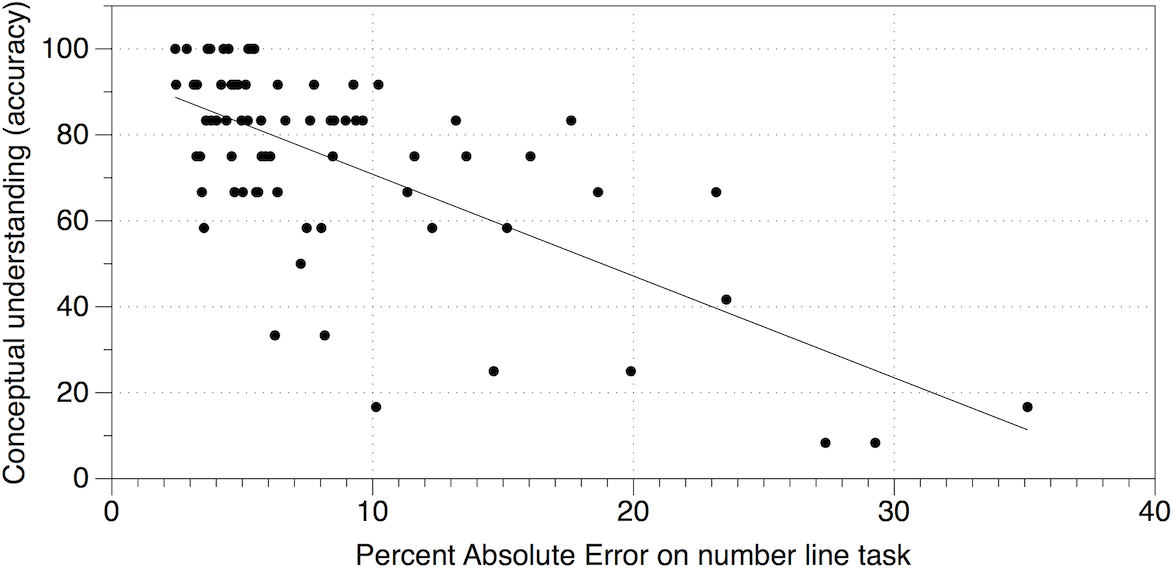
Scatterplot depicting the relationship between performance (percent absolute error) on the number line task and conceptual understanding (accuracy).

Model 1a (Table 4), demonstrates that, of the domain-general skills, working memory and inhibition were significant independent predictors of conceptual understanding. However, when the quantitative skills were added to the model in step 2, none of the domain-general skills remained significant predictors. This suggests that the association between working memory/inhibition and conceptual understanding may be mediated by basic quantitative skills, in particular number line performance. Of the quantitative skills, only number line performance was a significant independent predictor of conceptual understanding. This pattern is confirmed by Model 2a (Table 5). When the quantitative skills are added to the model first, number line performance is still the only independent predictor of conceptual understanding. Adding performance on the domain-general tasks does not significantly improve the fit of the model. In summary, from the wide range of basic arithmetic skills, numerical representations and domain-general skills assessed in this study, number line task performance emerges as the strongest, and only consistent, predictor of conceptual understanding. Children who made more accurate estimates of the position of numbers on the number line task performed more accurately on the conceptual understanding task (Fig 2).

The comparison models (Models 1b & 2b) confirm that this pattern is specific to conceptual understanding. When mathematics achievement was included as the dependent variable a different pattern of predictors was observed. Specifically, arithmetic strategy efficiency, inhibition and switching were significant independent predictors. Similar patterns were observed when the composite mathematics achievement measure was broken down in subtests (S2 Table); in both cases number line task performance was not a significant unique predictor. Thus it appears that number line task performance has a particular role in conceptual understanding.

### Exploring number line task performance

To explore the mechanisms that may be responsible for the relationship between number line performance and conceptual understanding, we conducted exploratory analyses of performance on the number line task. Previous research has suggested that children may employ a strategy on number line tasks based around using marker points such as the origin, end, midpoints and quartiles to guide their responses [61–62]. Similar strategies have also been observed with fraction number lines [51]. One explanation of the relationship between number line performance and conceptual understanding could be that children with better conceptual understanding make more use of these types of strategies. If this were the case then we would expect three patterns of results: 1) Children with better conceptual understanding should show greater accuracy for estimates close to marker points than children with lower levels of conceptual understanding, and this advantage should be greater than for estimates that are not close to marker points. 2) The difference between children’s accuracy for marker point estimates compared with other estimates should be positively correlated with conceptual understanding because children with better conceptual understanding would show a greater advantage for these strategic estimates. 3) Accuracy for marker point estimates should be associated with conceptual understanding over and above the relationship with accuracy for other estimates. We explored these possible patterns.

The number line task included five trials that were close to the origin, end, midpoints and quartiles of the 0–1000 line (2, 246, 486, 754, 938). We calculated two new measures of performance on the number line task: mean PAE for these 5 marker trials, and mean PAE for the remaining 17 non-marker trials. Overall performance was significantly more accurate for the marker trials (PAE M = 7.1, SD = 3.3) compared to the non-marker trials (PAE M = 9.1, SD = 8.1; *t*(75) = -2.81, *p* = .006), suggesting that children did employ this strategy to some extent.

**Fig 3:**
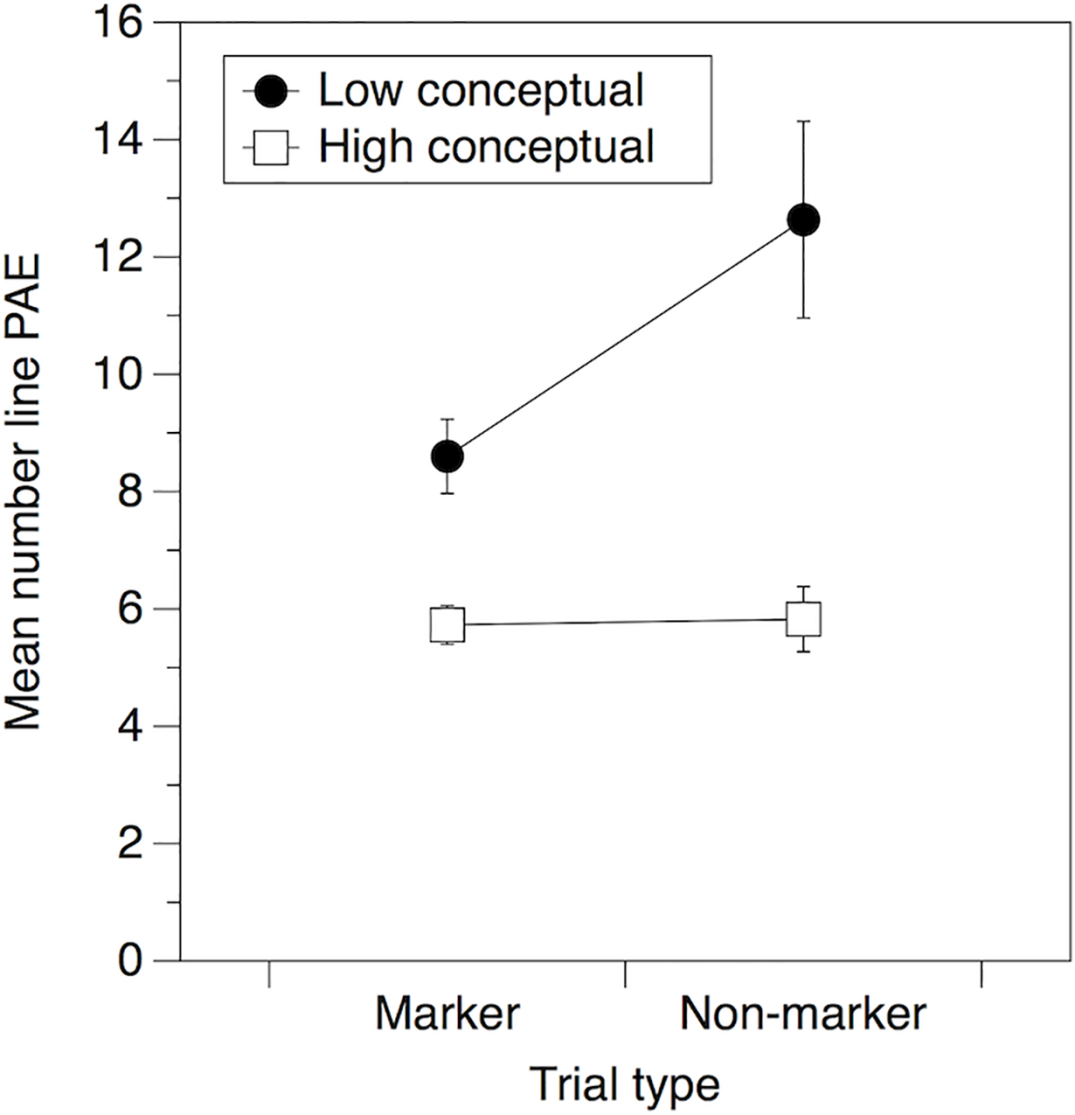
Mean percent absolute error (PAE) on number line trials which were either close to an endpoint or quartile (marker) or not (non-marker) as a function of conceptual understanding group. Error bars show standard error of the mean.

To investigate the first pattern, that children with better conceptual understanding would show a particular advantage for the marker trials, we used a median split on conceptual understanding scores to divide the sample into two groups; low and high conceptual understanding. We then conducted a mixed design ANOVA on PAE scores with group (high conceptual, low conceptual) as between-groups factor and trial type (marker, non-marker) as within-subject factor. This revealed a significant main effect of group, *F*(1,74) = 19.01, *p* < .001, *η*_*p*_^2^ = .20, trial type, *F*(1,74) = 9.66, *p* = .003, *η*_*p*_^2^ = .12, and a significant interaction between group and trial type, *F*(1,74) = 8.77, *p* = .004, *η*_*p*_^2^ = .11. As depicted in Fig 3, in contrast to the expected pattern, children with good conceptual understanding did not show a particular advantage for strategic trials. In fact, children with higher levels of conceptual understanding had no difference in performance for the marker (PAE = 5.73) and non-marker trials (PAE = 5.83), whereas children with lower levels of conceptual understanding were more accurate for marker, compared with non-marker trials (PAE = 8.60 and 12.63, respectively, *F*(1,35) = 9.68, *p* = .004, *η*_*p*_^2^ = .22). In line with this, and in contrast to the second expected pattern, there was a significant *negative* correlation between the difference in PAE for marker and non-marker trials and conceptual understanding (*r* = -.58, *p* < .001). Participants who had a greater difference in PAE for marker compared with non-marker trials, had lower scores on the conceptual understanding task. One explanation of this result is that children with higher levels of conceptual understanding show less evidence of using a strategy associated with the use of endpoints and quartiles than children with lower levels of conceptual understanding. Alternatively, children with good conceptual understanding may make use of the marker points but are also able to extend this strategy to non-marker trials, for example by identifying that 366 falls between the first quartile and midpoint.

Finally, to explore the third proposed pattern, we explored the correlation between performance on the marker and non-marker trials and conceptual understanding. There was a significant correlation between conceptual understanding and performance on both the marker (*r* = -.55, *p* < .001) and non-marker (*r* = -.65, *p* < .001) trials of the number line task. Partial correlations were then carried out to explore if the performance on the marker trials was significantly associated with conceptual understanding after controlling for performance on the non-marker trials. However, we in fact found the opposite pattern. When controlling for performance on the non-marker trials, there was no correlation between conceptual understanding and performance on the marker trials (*r* = -.084, *p* = .475). In contrast, when controlling for performance on the marker trials, the correlation between conceptual understanding and performance on the non-marker trials remained significant (*r* = -.420, *p* < .001). These findings together suggest that the relationship between number line performance and conceptual understanding is primarily driven by performance on the non-marker trials of the number line task.

## Discussion

In this study, we explored the cognitive skills associated with children’s conceptual understanding of arithmetic. Expanding on Geary’s [23] framework, we identified key quantitative and domain-general skills that were significantly related to conceptual understanding. However on multivariable analyses, performance on a number line task emerged as the strongest and only independent predictor.

This finding replicates and extends the study by Jordan et al. [43] Jordan and colleagues also identified number line task performance as the strongest predictor of conceptual understanding of fractions. Given that previous work has demonstrated that number line task performance is related to fractions knowledge more generally [51], it was possible that Jordan et al.’s findings resulted from the use of fractions as a domain, rather than a relationship with conceptual understanding per se. However, we have shown that number line task performance is also a strong predictor of conceptual understanding in another domain, namely whole-number arithmetic. Moreover, we found that this relationship was specific to conceptual understanding; number line performance was not a significant independent predictor of mathematical achievement whether this was measured by an overall composite or by specific subtests.

There are at least three possible mechanisms that might explain the relationship between number line task performance and conceptual understanding. To some extent, these potential mechanisms reflect differences in interpretations of number line task performance that are emerging in the literature.

First, number line task performance may reflect the nature of underlying representations of magnitude [63] and better conceptual understanding may therefore be linked with more accurate magnitude representations. However, studies have cast doubt that number line task performance is driven entirely by underlying magnitude representations [64–65]. Furthermore, if the link with conceptual understanding was driven by underlying magnitude representations, we would also expect conceptual understanding to be associated with other measures of magnitude representations included in the present study, namely the non-symbolic and symbolic magnitude comparison tasks. However, there were only weak correlations between performance on these magnitude comparison tasks and conceptual understanding, and they were not significant independent predictors of conceptual understanding. Therefore, we do not believe that the nature of underlying numerical representations directly drives the relationship between number line task performance and conceptual understanding.

Secondly, performance on the number line task may reflect the use of particular strategies, such as using midpoints and quartiles [51, 61–62], and the relationship with conceptual understanding may therefore be driven by individual differences in children’s ability to identify and apply strategic approaches to problem solving. Indeed, the conceptual understanding task also required children to identify and use conceptually based strategies for solving arithmetic problems. It is thus plausible that the relationship with number line task performance reflects individual differences in the application of this type of strategic behaviour in general. We explored this potential mechanism by identifying particular marker trials of the number line task for which performance would be expected to be particularly accurate if children were using such a strategic approach. If the relationship with conceptual understanding was driven by strategic behaviour, then children who made more use of a marker-point strategy, and thus had a bigger difference in performance on strategic and non-strategic trials, would have better conceptual understanding. In fact, we found the opposite pattern. Children with bigger differences between performance on the marker and non-marker trials had lower levels of conceptual understanding. There are two possible interpretations of these findings. Children with better conceptual understanding may have made less use of a marker-point strategy than children with poorer conceptual understanding. Alternatively, children with good conceptual understanding did use a marker-point strategy, but were also able to use marker points to help them position non-marker quantities more accurately on the number line, perhaps via better understanding of the numerical relationship between marker and non-marker quantities. Either way, we found that the relationship between conceptual understanding and number line performance was primarily driven by performance on the non-marker trials.

Finally, therefore, the relationship between number line task performance and conceptual understanding may be driven by children’s understanding of the structure of the number system. Our conceptual understanding task required children to identify relationships including commutativity, the inverse relationship between addition and subtraction, and the successor principle. These concepts are all elements of additive composition–the principle that any natural number is the sum of other natural numbers, in other words, numbers are composed of other smaller numbers. This principle underlies the ordinal structure of the symbolic number system and understanding this structure is also a requirement of successful performance on number line tasks. Indeed, research has highlighted that number line task performance reflects children’s understanding of the number system [66]. Therefore, it is plausible that understanding the structure of the symbolic number system is the mechanism underlying the relationship between number line performance and conceptual understanding found here. Our findings regarding the importance of performance on non-marker trials would support this suggestion. Understanding number system structure is also important for conceptual understanding of fractions [51] and thus this mechanism would also explain the relationship between number line task performance and conceptual understanding within the domain of fractions as found by Jordan et al. [43]. Similarly, measures of ordinality have recently been found to be important predictors of overall mathematics achievement [67], and to mediate the relationship between magnitude representations and mathematics performance [68]. Further research should explore whether measures of ordinality can account for the relationship between number line performance and conceptual understanding. Siegler, Thompson and Schneider [51] found that children used two strategies on fraction number line tasks. As well as a midpoint strategy they found that children made use of a transformation strategy which involved drawing on number system knowledge to convert the fraction into a more convenient number. This process requires knowledge of the structure of the rational number system. Children may similarly draw on knowledge of the number system to solve whole-number number line tasks and thus performance on number line tasks may be related to other tasks which required this knowledge (i.e. the conceptual understanding task used here).

The data in this study are cross-sectional and we therefore cannot draw conclusions regarding the direction of the relationship between number line task performance and conceptual understanding. If, as we suggest, knowledge of the structure of the number system does drive performance on both of these tasks then it is not meaningful to suggest that either better number line estimation helps children develop better conceptual understanding or vice-versa. We know that both number line estimation skills and basic understanding of additive composition can emerge from at least age 5 [4, 24]. It is possible therefore that these skills develop at the same time, driven by developments in understanding of the structure of the symbolic number system.

Beyond number line task performance, we explored the role of a broad range of quantitative and domain-general skills in explaining individual differences in conceptual understanding. Although we found significant zero-order correlations between conceptual understanding and quantitative skills such as counting, number fact knowledge and strategy use and efficiency, none of these correlations represented independent relationships, once number line performance was taken into account. The lack of strong relationships between conceptual understanding and quantitative skills provides further evidence that conceptual understanding and procedural skills are separable components of mathematics performance [19]. Consequently, it is unlikely that conceptual understanding of arithmetic arises simply from the development of, or instruction in, these basic quantitative skills. Rather, specific types of experiences and instruction may be needed to support the development of conceptual understanding [69–70].

Our study also included a broader range of cognitive measures of domain-general skills than previous studies [43]. We found that both working memory and inhibition were significant predictors of conceptual understanding, when quantitative skills were not added to the model. Previous research has found conflicting results concerning the relationship between working memory and conceptual understanding [42, 44] and between inhibition and conceptual understanding [45]. In contrast to previous studies, we did not find that the relationship with conceptual understanding varied according to the nature of the working memory task, either in terms of the distinction between verbal or visuospatial working memory, or according to whether or not the task included numerical stimuli.

Conflicting findings in the literature regarding the relationship between conceptual understanding and working memory and inhibition may be a consequence of different methods used to assess both conceptual understanding and inhibition. A wide variety of methods have been developed to assess conceptual understanding, including the application of procedures, the evaluation and justifications of procedures, or explicit descriptions of a concept [31]. It is likely that these different methods of assessment involve domain-general skills to varying extents. For example, inhibition may play a role when assessing conceptual understanding via the application of procedures, as used in this study, but not when assessing conceptual understanding through the identification of conceptual relationships [45].

We found that working memory was the strongest domain-general predictor of children’s use of conceptually-based strategies for solving problems but that working memory explained only a very small amount of variance in conceptual understanding once number line performance was taken into account, replicating Jordan et al. [43]. Previous research has found that working memory is significantly related to number line task performance [71]. Our analyses suggest that variance in working memory contributes to variance in both number line performance and conceptual understanding. Therefore, although conceptual understanding draws on domain-general skills, these are not unique to conceptual understanding but are also involved in lower-level numerical skills.

In this study visuo-spatial skills were not significantly related to conceptual understanding or mathematical achievement. This conflicts with previous research which has found relationships between visuo-spatial skills and both specific number skills and general measures of mathematical achievement [72, 73]. This contrast may be explained by our use of a single measure of visuo-spatial skills whereas previous studies have used more comprehensive measures.

The proportion of variance in conceptual understanding accounted for in this study is around 60%. Although this is less than the proportion of variance in mathematics achievement accounted for (84%), this figure is substantially higher than in previous investigations of conceptual understanding. For example, Cragg et al., [45] found only 5% of variance in conceptual understanding was accounted for by executive function skills compared to 34% of variance in overall mathematics achievement. The few studies to investigate cognitive correlates of conceptual understanding to date have focused on basic quantitative skills or executive functions. Language skills are also important predictors of general mathematics outcomes [74] and it is plausible that this may also play a role in conceptual understanding by allowing children to draw good understanding of mathematical language such as quantitative and spatial words [75]. Beyond cognitive skills, differences in pedagogy, for example the relative focus on procedural fluency vs. deeper problem solving, may also contribute to differences in children’s conceptual understanding of arithmetic. As outlined in the introduction, there are individual differences in children’s profile of performance across different components of mathematics and some children may have advanced conceptual understanding despite poorer procedural skills, while others show the opposite pattern. It would be valuable to explore how far differences in the cognitive and quantitative skills identified here can account for differences in children’s profile of performance across conceptual and procedural components of mathematics.

The strengths of this study lie in the inclusion of a broad range of cognitive measures of quantitative skills, numerical representations and domain-general skills, as well as the use of an established task for assessing conceptual understanding within a single study. Our data are from a single time point, however, and thus can only shed light on correlations with concurrent task performance. We do not know, for example, whether the skills identified here are also related to changes in conceptual understanding over time, and whether they represent causal relationships. This was an exploratory study and therefore the results should be replicated with pre-registered studies, with clearly stated hypotheses, to confirm these findings.

In conclusion, we have demonstrated that number line task performance accounts for variance in conceptual understanding of arithmetic, after a wide range of quantitative skills and domain-general skills have been taken into account. We hypothesize that the mechanism underlying this relationship is children’s understanding of the structure of the number system. This study adds to our understanding of the role of conceptual understanding in mathematics, and sheds light on the skills that may be involved in developing conceptual understanding. Activities to promote children’s understanding of the structure of the number system may therefore also help children to develop broader conceptual understanding of arithmetic.

## Supporting information

S1 Table Correlations between conceptual understanding, mathematics achievement and all quantitative and domain-general skills. (PDF)

S2 Table Hierarchical linear regression predicting WIAT Numerical Operations (Model 3a) and Mathematical Reasoning (Model 3b) subtests by quantitative and domain-general skills.
(PDF)
