# Supplementary material for: Understanding arithmetic concepts: The role of domain-specific and domain-general skills
Source: PLoS One. 2018 Sep 25;13(9):e0201724. doi: 10.1371/journal.pone.0201724 (PMC6155447; doi:10.1371/journal.pone.0201724)
Supplement: S1 Table — (PDF) [file pone.0201724.s001.pdf]

S1 Table: Correlations between conceptual understanding, mathematics achievement and all quantitative and domain-general skills.

Task	1	2	3	4	5	6	7	8	9	10	11	12	13
1. Conceptual understanding													
2. Mathematics achievement	.696**												
3. Counting	.592**	.758**											
4. Number fact	.603**	.795**	.633**										
5. Arithmetic strategy[a]	-.592**	-.704**	-.612**	-.671**									
6. Arithmetic efficiency	-.606**	-.751**	-.659**	-.683**	.669**								
7. Number recognition	-.546**	-.621**	-.535**	-.643**	.414**	.698**							
8. Number line	-.675**	-.672**	-.585**	-.548**	.699**	.744**	.548**						
9. Non-symbolic comparison	.315**	.312**	.393**	.284*	-.292**	-.381**	-.358**	-.321**					
10. Symbolic comparison	-.263*	-.458**	-.290*	-.490**	.179	.336**	.460**	.277*	.083				
11. Working memory	.463**	.669**	.529**	.484**	-.565**	-.503**	-.372**	-.504**	.382**	-.243*			
12. Inhibition	.391**	.426**	.411**	.299**	-.457**	-.336**	-.219	-.335**	.237*	-.128	.352**		
13. Switching	.327**	.316**	.230*	.180	-.272*	-.196	-.215	-.255*	.050	-.097	.333**	.175	
14. Visuo-spatial processing	.299**	.325**	.287*	.250*	-.337**	-.307**	-.278*	-.421**	.254*	-.141	.470**	.281*	.422**

Measures: Mathematics achievement raw score; counting % accuracy; number fact knowledge % accuracy; arithmetic strategy use % basic strategies; arithmetic efficiency RT; number recognition RT; number line PAE; non-symbolic comparison % accuracy; symbolic comparison RT; working memory composite score; Inhibition NEPSY-II standard score; switching NEPSY-II standard score; visuo-spatial processing NEPSY-II standard score. ** $p < .001$, * $p < .005$

[a] Correlation between percent % strategy use and accuracy on strategy task: $r = -.481$, $p < .001$
