# Supplementary material for: Understanding arithmetic concepts: The role of domain-specific and domain-general skills
Source: PLoS One. 2018 Sep 25;13(9):e0201724. doi: 10.1371/journal.pone.0201724 (PMC6155447; doi:10.1371/journal.pone.0201724)
Supplement: S2 Table — Hierarchical linear regression predicting WIAT Numerical Operations (Model 3a) and Mathematical Reasoning (Model 3b) subtests by quantitative and domain-general skills. (PDF) [file pone.0201724.s002.pdf]

S2 Table: Hierarchical linear regression predicting WIAT Numerical Operations (Model 3a) and Mathematical Reasoning (Model 3b) subtests by quantitative and domain-general skills.

		Model 3a DV: Numerical Operations			Model 3b DV: Mathematical Reasoning		
Step	Predictor	β	*t*	*p*	β	*t*	*p*
1	Age (months)	.284	2.551	.013	.207	1.817	.073
2	Age (months)	.234	2.423	018	.160	1.776	.080
	Working memory (composite)	.468	4.174	<.001	.492	4.702	<.001
	NEPSY-II Inhibition (SS)	.261	2.620	.011	.272	2.935	.005
	NEPSY-II Switching (SS)	.112	1.106	.272	.170	1.800	.076
	NEPSY-II Visuo-spatial processing (SS)	-.102	-.945	.348	-.042	-.417	.678
3	Age (months)	.072	.866	.390	.025	.357	.722
	Working memory (composite)	.219	2.323	.023	.229	2.845	.006
	NEPSY-II Inhibition (SS)	.097	1.215	.229	.096	1.404	.165
	NEPSY-II Switching (SS)	.067	.876	.384	.110	1.684	.097
	NEPSY-II Visuo-spatial processing (SS)	-.134	-1.608	.113	-.070	-.977	.333
	Counting (%)	.250	2.420	.018	.223	2.517	.014
	Number fact knowledge (%)	.435	3.536	.001	.202	1.916	.060
	Arithmetic strategy use (% basic)	.078	.594	.555	-.051	-.449	.655
	Arithmetic strategy efficiency (RT)	-.076	-.586	.560	-.178	-1.591	.117
	Number recognition (RT)	.139	1.302	.198	-.112	-1.222	.226
	Number line (PAE)	-.168	-1.382	.172	-.040	-.385	.701
	Non-symbolic comparison (%)	-.048	-.584	.561	-.056	-.791	.432
	Symbolic comparison (RT)	-.090	-1.053	.296	-.054	-.732	.467

Model 3a: R^2 change for Step 1 = .081, p = .013; R^2 change for Step 2 = .368, $p < .001$; R^2 change for Step 3 = .287, $p < .001$; Total R^2 = .735; Model 3b: R^2 change for Step 1 = .043, p = .073; R^2 change for Step 2 = .479, $p < .001$; R^2 change for Step 3 = .284, $p < .001$; Total R^2 = .806; RT = reaction time; PAE = Percent Absolute Error.
